# In Situ Reaction Induced Core–Shell Structure to Ultralow κ_lat_ and High Thermoelectric Performance of SnTe

**DOI:** 10.1002/advs.201903493

**Published:** 2020-04-16

**Authors:** Sihui Li, Jiwu Xin, Abdul Basit, Qiang Long, Suwei Li, Qinghui Jiang, Yubo Luo, Junyou Yang

**Affiliations:** ^1^ State Key Laboratory of Materials Processing and Die & Mould Technology Huazhong University of Science and Technology Wuhan 430074 P. R. China

**Keywords:** β‐Zn_4_Sb_3_, core–shell structures, in situ decomposition reaction, SnTe thermoelectric materials, tin telluride

## Abstract

Lead‐free chalcogenide SnTe has been demonstrated to be an efficient medium temperature thermoelectric (TE) material. However, high intrinsic Sn vacancies as well as high thermal conductivity devalue its performance. Here, β‐Zn_4_Sb_3_ is incorporated into the SnTe matrix to regulate the thermoelectric performance of SnTe. Sequential in situ reactions take place between the β‐Zn_4_Sb_3_ additive and SnTe matrix, and an interesting “core–shell” microstructure (Sb@ZnTe) is obtained; the composition of SnTe matrix is also tuned and thus Sn vacancies are compensated effectively. Benefitting from the synergistic effect of the in situ reactions, an ultralow κ_lat_ ≈0.48 W m^−1^ K^−1^ at 873 K is obtained and the carrier concentrations and electrical properties are also improved successfully. Finally, a maximum ZT ≈1.32, which increases by ≈220% over the pristine SnTe, is achieved in the SnTe‐1.5% β‐Zn_4_Sb_3_ sample at 873 K. This work provides a new strategy to regulate the TE performance of SnTe and also offers a new insight to other related thermoelectric materials.

## Graphical abstract

In situ chemical reactions occur between the β‐Zn_4_Sb_3_ additive and SnTe matrix, and the resultant interesting “core–shell” structure is obtained in this work. The composition, microstructure, and transport properties of SnTe thermoelectric materials are synergistically tuned, so an ultralow lattice thermal conductivity (≈0.48 Wm^−1^ K^−1^ at 873 K) and relatively high ZT (≈1.32 at 873 K) are achieved, which present an effective method to enhance the thermoelectric performance of SnTe, and are also of referential values for other thermoelectric materials.

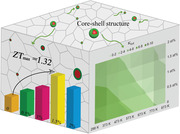

## 1 Introduction

Thermoelectric (TE) materials that can directly convert heat to electrical energy have gained wide attention due to global concerns regarding energy efficiency and conservation.^1^ TE conversion efficiency depends on the performance of TE materials denoted by the dimensionless TE figure of merit ZT, ZT = *S* ^2^ *σT*/(κ_e_ + κ_l_), where *S* is the Seebeck coefficient, σ is the electrical conductivity, κ_e_ is the electronic thermal conductivity, κ_l_ is the lattice thermal conductivity, and *T* is the absolute temperature, respectively.^2^ Notably, a good TE material with high ZT concurrently requires a large power factor (*S* ^2^σ) and low thermal conductivity.^3^

Lead chalcogenides PbQ (Q = Te, Se, S) with a rock‐salt structure, especially PbTe, have been demonstrated to be efficient medium temperature thermoelectric materials.^4^ However, the toxicity of lead devalues its thermoelectric applications. SnTe is a lead‐free analog of PbTe with a similar rock‐salt crystal structure and double valence band structure as PbTe and has been thought to be a promising candidate for TE applications.^5^ Unfortunately, it presents a low TE performance due to the intrinsically high carrier concentration, smaller bandgap as well as larger valence band offsets with respect to PbTe.^6^ By means of doping or alloying with various elements, the electrical performance of SnTe has been effectively boosted.^7^ For example, Zhou et al. reduced the carrier concentration and increased the Seebeck coefficient by Bi doping and obtained a maximum ZT value of 1.1 at 873 K in Sn_0.94_Bi_0.06_Te sample.^8^ Tan et al. optimized the band structure of SnTe and realized a valence band convergence and achieved a high Seebeck coefficient by Cd, Hg, and Mn alloying.^9^ Although the ZT of SnTe has been much improved by augmenting the power factor, there is less room for further enhancement unless thermal conductivity could be much decreased simultaneously. Recently, Biswas and co‐workers reported a ZT ≈1.0 by reducing the lattice thermal conductivity (κ_lat_) near the theoretical minimum limit of SnTe via Sb rich nanoprecipitates along with super‐structured intergrowth nanodomains derived from Sb doping.^10^ Pei and co‐workers obtained a high ZT value to ≈1.4 through reducing the κ_lat_ close to the amorphous limit by strong phonon scattering of Cu_2_Te nanoprecipitates as well as Cu interstitials.^11^ Obviously, the reduction in thermal conductivity is equally and even more effective in enhancing the ZT value of SnTe.^12^

β‐Zn_4_Sb_3_ is a well‐known and eco‐benign p‐type compound. Our previous work demonstrated that it could remarkably reduce the phonon thermal conductivity and enhance the thermoelectric performance of p‐type (Bi,Sb)_2_Te_3_‐based materials by decomposing into Zn and ZnSb nanoinclusions.^13^ Based on this consideration, herein, β‐Zn_4_Sb_3_ was incorporated into the matrix of SnTe to improve its thermoelectric performance. Very unexpectedly and interestingly, a special “core–shell” structure with Sb core and ZnTe shell (Sb@ZnTe) has been obtained. Benefitting from the in situ reaction and the associated “core–shell” microstructure, an ultralow κ_lat_ ≈ 0.48 W m^−1^ K^−1^ has been achieved while a relatively high power factor has also been maintained and thus a high thermoelectric performance with ZT of ≈1.32 has been obtained at 873 K for the SnTe‐1.5% β‐Zn_4_Sb_3_ sample. Notably, single doping and co‐doping with Zn and Sb have also been studied for comparison, and the results illustrated that only the β‐Zn_4_Sb_3_ containing samples show special “core–shell” microstructures and achieved higher TE performance. This work presents an effective method to enhance the TE performance of SnTe, which also offers a new insight to other related thermoelectric materials.

## 2 Results and Discussion

### 2.1 In Situ Decomposition Reaction and Core–Shell Structure

**Figure 1:**
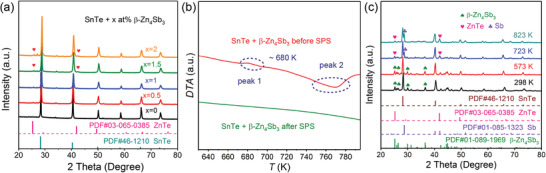
a) X‐ray diffraction (XRD) patterns of the samples with different content of β‐Zn_4_Sb_3_ additives; b) differential thermal analysis (DTA) curves of the powder mixture of SnTe‐2% β‐Zn_4_Sb_3_ before and after SPS; c) in situ XRD patterns of the SnTe‐10% β‐Zn_4_Sb_3_ powder mixture at different temperatures.

**Figure** **1**a depicts the X‐ray diffraction (XRD) patterns of the SnTe‐*x* at% β‐Zn_4_Sb_3_ (*x* = 0, 0.5, 1, 1.5, and 2) samples. The main peaks in all patterns can be well indexed to the cubic SnTe structure (PDF# 46‐1210). Besides that, blende ZnTe phase (PDF#03‐065‐0385) also shows up when the content of β‐Zn_4_Sb_3_ is higher than 1 at%. No peak of β‐Zn_4_Sb_3_ can be found in the XRD patterns, indicating that all the β‐Zn_4_Sb_3_ nanopowders have been decomposed during the process of spark plasma sintering (SPS) as consistent with the reported work,^14^ the decomposition reaction is as follows

(1) $$\beta {\text{-Zn}}_{4}{\text{Sb}}_{3}\to \text{Zn}+3\;\text{ZnSb}$$

From this reaction, the decomposition products are Zn and ZnSb, but why is there small amount of ZnTe phase but not ZnSb phase appear in the above XRD patterns? The differential thermal analysis (DTA) result in Figure 1b gives the answer. There are two peaks in the DTA curve of the SnTe + β‐Zn_4_Sb_3_ powder mixture before SPS, the first endothermic peak around ≈680 K corresponds to the decomposition temperature of β‐Zn_4_Sb_3_, and the second wide exothermic peak around 770 K indicates that another reaction was happened between the decomposition products and the SnTe matrix at elevated temperature, which should be ascribed to the formation of ZnTe phase, as depicted by the following equation

(2) $$\text{SnTe}+x\;\text{Zn}+3x\;\text{ZnSb}\to \;3x\;\text{Sb}@\text{ZnTe}+{\text{Sn}}_{1-4x}{\text{Zn}}_{x}{\text{Te}}_{1-3x}+4x\;\text{Sn}$$

This reaction can be verified by the in situ high temperature XRD measurement as shown in Figure 1c. As can be seen, the disappearance of β‐Zn_4_Sb_3_ accompanies with the appearance of ZnTe and Sb phase once the temperature is over 723 K, which is in good agreement with the DTA results. It should be noted here that the sample with high content of 10% β‐Zn_4_Sb_3_ is chosen so that the reaction products can be well distinguished in the high temperature in situ XRD patterns, considering no peak of Sb is observed in the SPS samples (Figure 1a) due to the detection limit. As for the special Sb@ZnTe microstructure, it will be further discussed in the following part.

**Figure 2:**
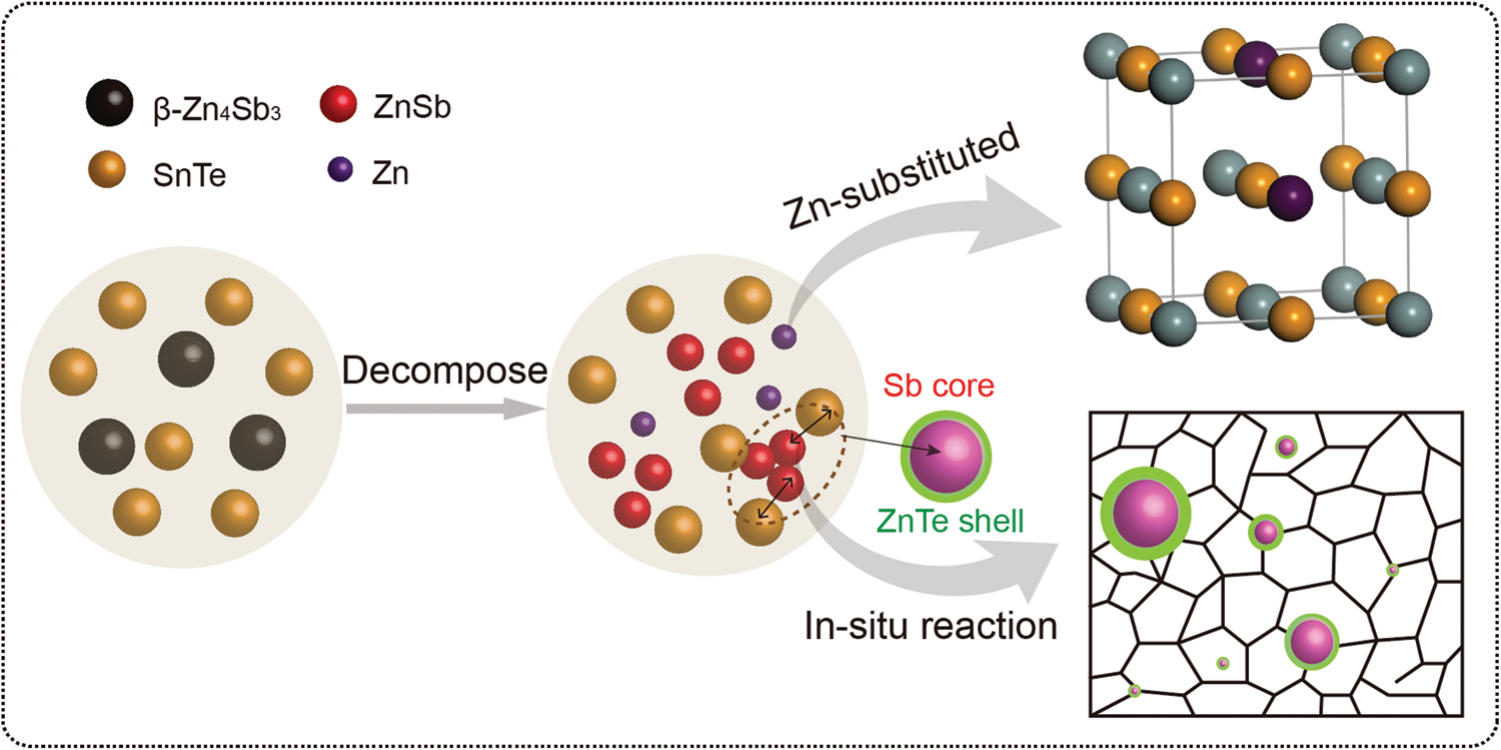
The schematic diagram of the reaction process of the “core–shell” structure.

**Figure** **2** shows the schematic diagram for the reactions, which mainly include two processes as mentioned above: the first is the thermal decomposition of β‐Zn_4_Sb_3_, which results in the products of Zn‐substituted SnTe solid solution and ZnSb compounds, then the ZnSb compounds will further react with the SnTe matrix, and forms the Sb@ZnTe special “core–shell” structure. The mechanism behind this reaction can be illustrated in the perspective of chemical bonding; it is known that the electronegativity of Te is stronger than Sb, while the metallicity of Zn is stronger than that of Sn, so the chemical bond between Zn and Te is the strongest among the bonds of Zn, Sn, Sb elements with Te. And the calculated Gibbs free energy (−44.5 kJ mol^−1^), as listed in Table S1 in the Supporting Information, further confirms that the reaction is favorable in thermodynamics.^15^ Therefore, during the SPS process, the Zn^2+^ in ZnSb nanoparticles (NP) gradually combines with Te^2−^ in SnTe matrix and results in the formation of the Sb@ZnTe core–shell structures, which is significantly conducive to the TE performance of SnTe and will be detailed below.

### 2.2 Microstructure Characterization

**Figure 3:**
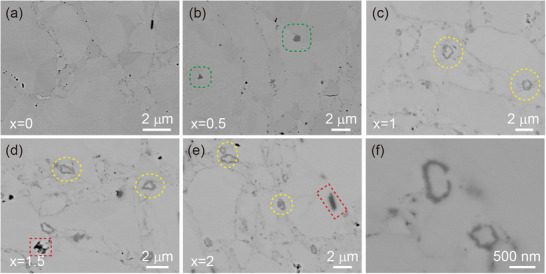
Backscattering scanning electron (BSE) images of a) the pristine SnTe and b–e) SnTe‐*x* at% (*x* = 0.5, 1, 1.5, and 2) β‐Zn_4_Sb_3_ samples; f) magnified image of the SnTe‐2% Zn_4_Sb_3_ sample.

**Figure** **3** shows the backscattering scanning electron (BSE) images of the pristine SnTe and SnTe‐*x*% β‐Zn_4_Sb_3_ samples, respectively. It can be seen that the pristine SnTe matrix demonstrates typical polycrystalline morphology (Figure 3a). When a small amount of β‐Zn_4_Sb_3_ was added, some dark gray nanoprecipitates appeared (green area, Figure 3b) due to the solid solution limit. The point element analysis in Figure S2a in the Supporting Information shows that the chemical composition of the nanoprecipitates is ZnTe. As the content of β‐Zn_4_Sb_3_ increases, some special “core–shell” structures with size of ≈1 µm or hundreds of nanometers can be clearly seen in Figure 3c–f. Besides, another new precipitates (red area) also can be observed in the high content samples (Figure 3d,e), which can be confirmed as Sn phase by the electron probe microanalysis (EPMA) results in Figure S2 in the Supporting Information.

**Figure 4:**
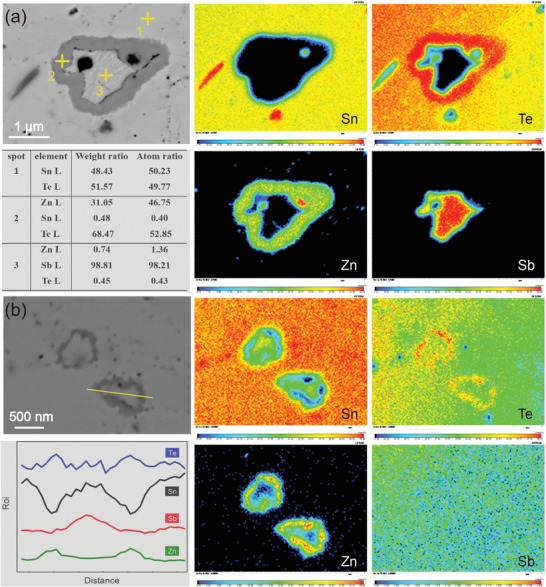
Electron probe microanalysis (EPMA). Element analysis of SnTe‐1.5% β‐Zn_4_Sb_3_ samples.

To further figure out the composition of the special “core–shell” microstructure, **Figure** **4** gives the EPMA element mapping results of the SnTe‐1.5% β‐Zn_4_Sb_3_ sample. For more meticulous characterization of the particular structure, a relatively large size “core–shell” structure was selected (Figure 4a), and the element mapping results demonstrated that the chemical compositions of the shell agree well with ZnTe and the core is Sb element, which can be confirmed by the point composition analysis results from spot 1 to spot 3. Besides, the Sn phase also can be observed in the red box area in Figure 4a. In addition, there are many small nanoscale core–shell particles, as shown in Figure 3b, and the EPMA element mapping and line scanning results also verify the same “core–shell” structure with ZnTe shell and Sb core.

**Figure 5:**
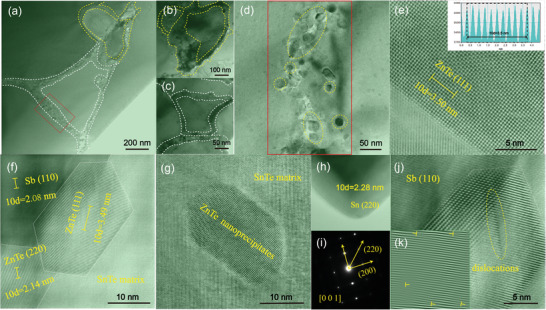
a–c) Low and medium magnification transmission electron microscope (TEM) images of core–shell structure; d) magnified TEM image of the red area; high resolution TEM (HRTEM) image of e) the nanoparticles, f) core–shell structure, and g,h) nanoprecipitates; i) IFFT image of the SnTe matrix; j) HRTEM image of the point defects and dislocations; and k) DFFT image of the dislocations.

Further, transmission electron microscope (TEM) was employed to characterize the core–shell microstructure and the results are shown in **Figure** **5**. Figure 5a–c depict the TEM images of specific “core–shell” microstructure, and it can be seen more clearly in the magnified image (Figure 5d), it shows that the shell (red area) is actually constituted with numerous tightly aligned nanoparticles with a size of ≈20 nm, which exhibit a typical ZnTe (111) plane in the high resolution image (Figure 5e), just in good consistence with the EPMA results. Figure 5f demonstrates the high‐resolution transmission electron microscope (HRTEM) image of the core area with a typical Sb (110) plane and the arranged ZnTe nanoparticles shell. Besides, because of the low solid solubility, there are also some separate ZnTe nanoprecipitates as well as Sn phase appeared in the matrix (Figure 5g,h), and Figure 5i demonstrates the inverse fast Fourier transform (IFFT) image of SnTe matrix. In addition, point defects (PD) and dislocations were also observed in the sample, as shown in Figure 5j,k. All these hierarchical microstructure will cooperatively contribute to multiscale phonon scattering and thus remarkably reduce the lattice thermal conductivity.

### 2.3 Thermoelectric Properties

**Figure 6:**
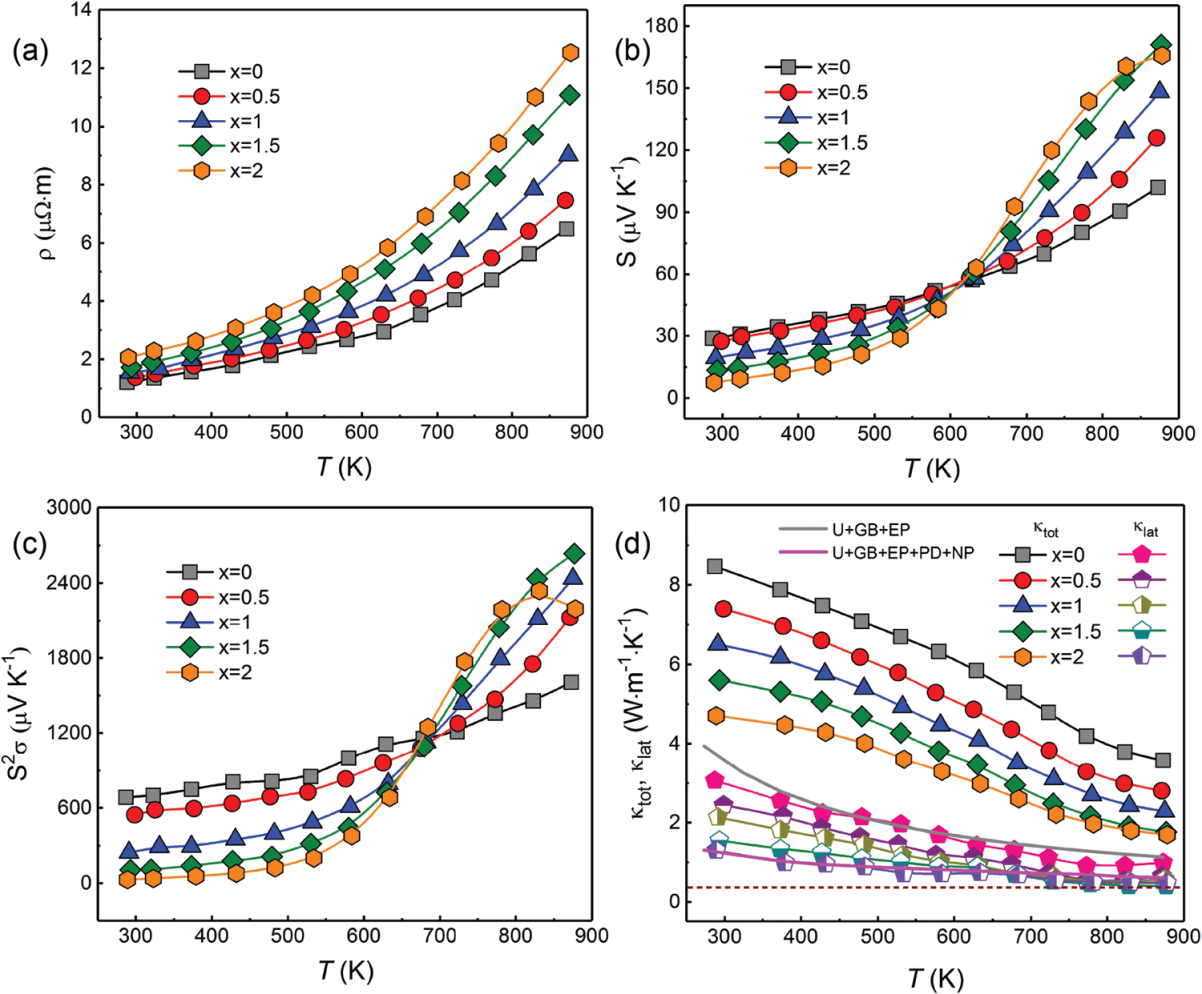
a) Temperature dependent electrical resistivity; b) Seebeck coefficient; c) power factor; and d) total thermal conductivity and lattice thermal conductivity for the SnTe + *x* at% β‐Zn_4_Sb_3_ (*x* = 0, 0.5, 1, 1.5, and 2) samples.

**Figure** **6**a presents the temperature dependent thermoelectric properties for the SnTe‐*x* at% β‐Zn_4_Sb_3_ (*x* = 0, 0.5, 1, 1.5, and 2) samples. We can see that the resistivities (ρ) increase with increasing β‐Zn_4_Sb_3_ content, which can be illustrated by the carrier concentration reduction as listed in **Table** **1**. We can see that the carrier concentration (*n*_H_) of the pristine SnTe is ≈1.36 × 10^20^ cm^−3^ and gradually decreases to ≈7.4 × 10^19^ cm^−3^ for the sample with 2% β‐Zn_4_Sb_3_, which should be ascribed to the substitution Zn for Sn and the resultant Sn self‐compensation effect by the in situ reaction mentioned above.

**Table 1:** Carrier concentration (*n*_H_) and Hall mobility (μ_H_) for the SnTe‐*x*% β‐Zn_4_Sb_3_ (*x* = 0, 0.5, 1, 1.5, and 2) samples at room temperature

Samples	*n*_H_ [10^19^ cm^−3^]	μ_H_ [cm^2^ V^−1^ s^−1^]
SnTe	13.6	379.8
SnTe‐0.5% β‐Zn_4_Sb_3_	10.51	399.1
SnTe‐1% β‐Zn_4_Sb_3_	8.97	374.6
SnTe‐1.5% β‐Zn_4_Sb_3_	7.94	342.4
SnTe‐2% β‐Zn_4_Sb_3_	7.4	315.3

However, the Seebeck coefficient, *S*, as shown in Figure 6b, decreases gradually with the increase of β‐Zn_4_Sb_3_ content in low temperature range, and then increases significantly with the content of β‐Zn_4_Sb_3_ when the temperature is over 600 K. This anomalous phenomenon should be attributed to the decrease of Sn vacancies caused by Zn alloying and Sn self‐compensation, which is consistent with the previous reported work.^16^ As is well known, pristine SnTe has a very high hole concentration because of its excessive Sn vacancies, and the Fermi level is lower in energy and closer to the heavy‐hole band. With the decreasing hole concentration caused by Zn substitution and Sn self‐compensation effect resulted by the in situ reactions between β‐Zn_4_Sb_3_ and the matrix, the Fermi level rises and gradually shifts away from the heavy valence band (Figure S3, Supporting Information), only left the light‐hole band contributes to the Seebeck coefficient and thus results in a lower Seebeck value at low temperature range.^17^ However, when the temperature increases, the contribution of the heavy hole band increases due to the thermal excitations, both the light‐hole band and heavy‐hole band participate in the transport process, thus the effective mass increases to approach or exceed that of the matrix sample, hence the Seebeck coefficient increases at high temperature.^18^ Additionally, extra Zn alloying induced by the in situ reaction will also enhance the electrical performance of SnTe due to the band engineering, which has been verified in several reported work.^18^, ^19^ Combined with these multiple effects, the power factor of the SnTe‐*x* at% β‐Zn_4_Sb_3_ samples (Figure 6c) are greatly enhanced in high temperature range with respect to the pristine SnTe.

The total thermal conductivity (κ_tot_) shown in Figure 6d decreases monotonically with increasing *x* value. The κ_tot_ is essentially a combination of two parts: the electronic contribution (κ_el_) and the lattice contribution (κ_lat_).^20^ The electronic contribution can be evaluated by the Wiedemann−Franz relation κ_el_ = *LσT*, where *L* is the Lorenz number, σ is the electrical conductivity, and *T* is the absolute temperature.^21^ The temperature dependent Lorenz number and κ_el_ can be found in Figure S4 in the Supporting Information. The κ_lat_ is then calculated by subtracting κ_el_ from κ_tot_, and the results are shown in Figure 6d. It can be seen that the κ_lat_ decreases with the content of β‐Zn_4_Sb_3_ and the minimum κ_lat_ is only about 0.48 Wm^−1^ K^−1^ at 873 K for the SnTe‐2 at% β‐Zn_4_Sb_3_ sample, which is close to the amorphous limit.^22^

**Figure 7:**
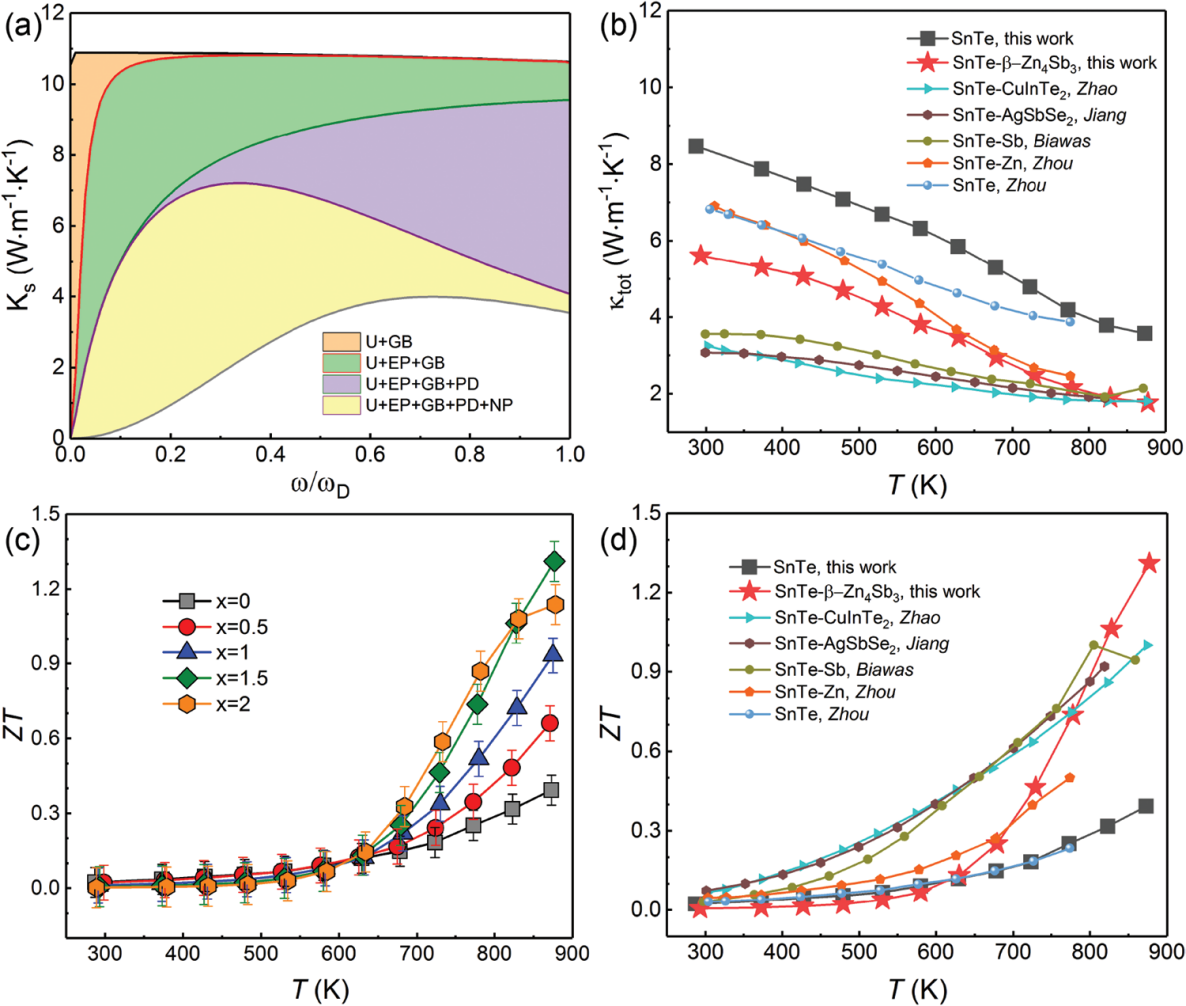
a) The calculated spectral lattice thermal conductivity (κ_s_) with different phonon scattering mechanisms of the SnTe‐1.5% β‐Zn_4_Sb_3_ at 873 K; b) a comparison of κ_tot_ for the SnTe‐1.5% β‐Zn_4_Sb_3_ with some relevant reported results;[qv: 10,19b,26] c) ZT value as a function of temperature for SnTe‐*x*% β‐Zn_4_Sb_3_ (*x* = 0, 0.5, 1, 1.5, and 2) samples; and d) ZT values comparison between SnTe‐1.5% β‐Zn_4_Sb_3_ sample in this work with other reported work.

The κ_s_ with respect to the phonon frequency (ω) has been calculated and presented in **Figure** **7**a to further understand of the origin of low κ_lat_.^23^ The U, GB, EP, PD, and NP represent the Umklapp process, grain boundary scattering, electron phonon scattering, point defect scattering, and nanoparticles scattering, respectively.^24^ It can be seen that the NP and point defects produced by the in situ decomposition reaction mainly enable an extra strong scattering to the medium and high frequency phonons, which play a major role in the reduction of κ_lat_. In addition, the κ_lat_ was also calculated according to the well‐known Callaway model (refer to the Supporting Information) with consideration of all these hierarchical factors (U+EP+GB+PD+NP) in the SnTe‐β‐Zn_4_Sb_3_ sample as a reference to SnTe matrix (Figure 6d).^24^, ^25^ One can easily see that the theoretical model agrees well with the experimental curves, which implies that the multiscale microstructures induced by addition of β‐Zn_4_Sb_3_ can significantly enhance phonons scattering and thus result in an ultralow κ_lat_. Combined with the reduced κ_el_, the κ_tot_ was greatly decreased compared with the pristine SnTe, and shows a relative low value at high temperature compared with several reported works, as shown in Figure 7b.

Benefitting from the low κ_lat_ and relatively high power factor, the ZT value of SnTe‐*x*% β‐Zn_4_Sb_3_ (*x* = 0, 0.5, 1, 1.5, and 2) samples were effectively enhanced (Figure 7c), and the maximum ZT reaches ≈1.32 at 873 K in the sample with *x* = 1.5, which increases by ≈220% improvement over the pristine SnTe. In addition, a comparison was also made in Figure 7d between the ZT value of this work and some relevant works with similar strategy by addition of secondary phase. It can be seen that the sample with β‐Zn_4_Sb_3_ achieves a remarkable enhancement of the thermoelectric performance of SnTe, further suggesting that the in situ chemical reaction strategy is more effective in enhancing thermoelectric performance of SnTe.

**Figure 8:**
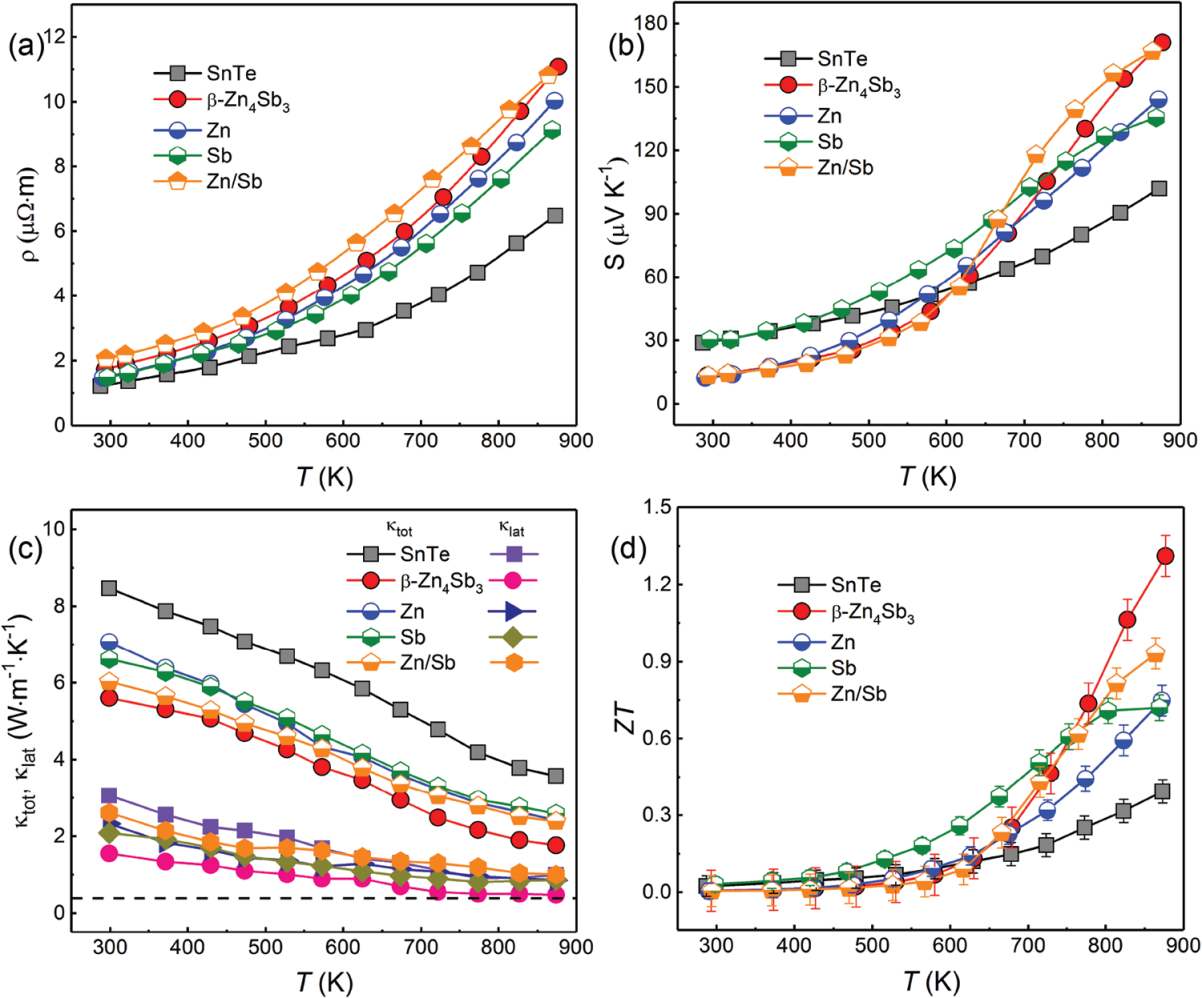
a) Temperature dependent electrical resistivity; b) Seebeck coefficient; c) total thermal conductivity and lattice thermal conductivity; d) ZT values for the pristine SnTe, SnTe‐1.5% β‐Zn_4_Sb_3_, SnTe‐6% Zn, SnTe‐4.5% Sb, and SnTe‐6% Zn‐4.5% Sb, respectively.

In order to systematically study the in situ reaction mechanism, the Zn‐doped SnTe, Sb‐doped SnTe, and Zn/Sb co‐doped SnTe samples were also fabricated and their thermoelectric properties were also studied and presented in Figures S5–S8 in the Supporting Information. **Figure** **8** shows a comparison of the thermoelectric performance of the SnTe‐1.5% β‐Zn_4_Sb_3_ sample with the corresponding single doped and co‐doped samples. As can be seen, the SnTe‐1.5% β‐Zn_4_Sb_3_ sample exhibits comparable electrical properties with the Zn/Sb co‐doped sample, and the Zn‐doped and Sb‐doped samples are inferior to the sample with addition of 1.5% β‐Zn_4_Sb_3_ in electrical performance (Figure 8a,b). Moreover, both the total and lattice thermal conductivities of SnTe‐1.5% β‐Zn_4_Sb_3_ sample, as demonstrated in Figure 8c, are much lower than those of the Zn‐, Sb‐ and Zn/Sb co‐doped samples, thus resulting in a much higher ZT value at high temperature (Figure 8d).

To reveal the reason behind the difference in thermal conductivity, EPMA element mapping analysis has been carried out to the Zn‐doped SnTe, Sb‐doped SnTe and Zn/Sb co‐doped SnTe samples, and the results are present in Figures S9–S11 in the Supporting Information. As can be seen, there are only several ZnTe and Sb nanoprecipitates, but no “core–shell” microstructure appears in these single and co‐doped samples. That is to say, the special “core–shell” microstructure, which should be attributed to the significant reduction of thermal conductivity, only exists in the samples with addition of β‐Zn_4_Sb_3_. That is why the β‐Zn_4_Sb_3_ containing samples show lower thermal conductivity and approximately approaches the amorphous limit in κ_lat_ with respect to other doped samples. The in situ decomposition reaction and the resultant “core–shell” microstructure play a significant role in reducing the thermal conductivity thus high TE performance of SnTe and also pave a new route and open new possibility to the improvement of TE performance of other thermoelectric materials.

## 3 Conclusions

In summary, by means of the in situ chemical reactions between the β‐Zn_4_Sb_3_ additives and SnTe matrix, a typical “core–shell” microstructure of Sb@ZnTe has been introduced to the matrix for the first time, the composition of SnTe matrix has been tuned, and thus Sn vacancies have been compensated effectively. Benefitting from the synergistic effect of the in situ reactions, the thermal conductivity especially lattice thermal conductivity has been reduced drastically, and the carrier concentrations and electrical properties have also been improved successfully. Finally, a maximum ZT value ≈1.32, which increases by ≈220% over the pristine SnTe, has been achieved in the SnTe‐1.5% β‐Zn_4_Sb_3_ sample at 873 K. This work provides a new route to the regulation of composition, microstructure and TE performance of SnTe, and is also of referential value for other thermoelectric materials.

## 4 Experimental Section

##### β‐Zn_4_Sb_3_ Compound Synthesis

High purity Zn shots (99.99 wt%) and Sb powders (99.99 wt%) were weighed in the stoichiometric molar ratio, and sealed in an evacuated quartz tube, then melted at 1023 K for 24 h and quenched in cool water.^13^ The obtained ingot was crushed and ball milled at 300 rpm for 1 h to gain fine powders. The powder XRD pattern is presented in Figure S1 in the Supporting Information, which shows a single β‐Zn_4_Sb_3_ phase.

##### Synthesis of SnTe and β‐Zn_4_Sb_3_ Composite

First, the SnTe was synthesized by melting the stoichiometric elemental mixture (Sn powder, 99.99 wt%, Te powder, 99.99 wt%, Aladdin, China) in sealed quartz tubes (10^−3^ Pa) at 1123 K for 10 h, soaked for 12 h, and quenched to room temperature. Then the as‐melt ingots were crushed and ball milled into fine powders. After that, different content of β‐Zn_4_Sb_3_ powders were added to the SnTe powders (SnTe‐*x* at% β‐Zn_4_Sb_3_, *x* = 0, 0.5, 1, 1.5, and 2) the powders were further mixed by ball milling at 250 rpm for 1 h. Finally, the powder mixtures were sintered and densified by SPS at 773 K for 10 min under a pressure of 60 MPa in a vacuum atmosphere.

##### Synthesis of Zn/Sb Single Doped and Co‐Doped SnTe Samples

For comparison, the corresponding content of Zn‐doped, Sb‐doped, and Zn/Sb co‐doped SnTe samples were also synthesized. High purity Zn shot and Sb powder were weighed in the stoichiometric molar ratio (SnTe‐*y*% Zn, *y* = 0, 2, 4, 6, and 8; SnTe‐*z*% Sb, *z* = 0, 1.5, 3, 4.5, and 6; SnTe‐*w*% Zn/Sb, *w* = 0, 2/1.5, 4/3, 6/4.5 and 8/6), and the mixture powders were sealed in quartz tubes and sintered with the same methods mentioned above. The relative density of all the obtained samples was above 95%.

##### Characterization

The phase characterization was analyzed by powder X‐ray diffraction patterns (PANalytical X'pert PRO diffractometer, with Cu Kα radiation, λ = 1.5406 Å). Field‐emission scanning electron microscope (NanoSEM 450), HRTEM, and energy‐dispersive X‐ray spectroscopy (EDS) observation of the selected specimens was performed with a JEOL JEM‐2100 transmission electron microscope. The DTA measurements were analyzed by using an STA449F3 (NETZSCH) equipment. The Seebeck coefficient, *S* and electrical resistivity (ρ) were simultaneously obtained by using a Namicro‐III thermoelectric measurement system. The measurement uncertainties typically are 5% for the Seebeck coefficient and 3% for the electrical resistivity. The Hall coefficient (*R*_H_) was measured by the van der Pauw method with a HMS‐5500 Hall measurement system. Carrier density (*n*_H_) and mobility (μ_H_) were calculated according to the relationship *n*_H_ = 1/(*e R*_H_) and μ_H_ = *σ R*_H_, where σ is the electrical conductivity obtained from the Namicro‐III system. The thermal conductivity was calculated via κ = *D* Cp ρ, where the thermal diffusivity coefficient (*D*) was measured with a NETZSCH LFA‐427 laser thermal conductivity instrument and the heat capacity (Cp) was determined by the empirical formula Cp (*k*_B_ per atom) = (3.07 + 0.00047 (T‐300)),^27^ and the relative densities (ρ) of the bulk samples were measured by the Archimedes principle.

## Conflict of Interest

The authors declare no conflict of interest.

## Supporting information

Supporting Information
