# Supplementary material for: In Situ Reaction Induced Core–Shell Structure to Ultralow κlat and High Thermoelectric Performance of SnTe
Source: Adv Sci (Weinh). 2020 Apr 16;7(11):1903493. doi: 10.1002/advs.201903493 (PMC7284213; doi:10.1002/advs.201903493)
Supplement: Supplementary file 1 — Supporting Information [file ADVS-7-1903493-s001.pdf]

Copyright WILEY-VCH Verlag GmbH & Co. KGaA, 69469 Weinheim, Germany, 2020.

ADVANCED SCIENCE Open Access

Supporting Information

for *Adv. Sci.,* DOI: 10.1002/advs.201903493

In Situ Reaction Induced Core–Shell Structure to Ultralow $\#_{lat}$ and High Thermoelectric Performance of SnTe

*Sihui Li, Jiwu Xin, Abdul Basit, Qiang Long, Suwei Li, Qinghui Jiang, Yubo Luo, and Junyou Yang**

Supporting Information

In-situ Reaction Induced Core-shell Structure to Ultralow κ_{lat} and High Thermoelectric Performance of SnTe

Sihui Li, Jiwu Xin, Abdul Basit, Qiang Long, Suwei Li, Qinghui Jiang, Yubo Luo and Junyou Yang*

* To whom correspondence should be addressed. Email: jyyang@mail.hust.edu.cn

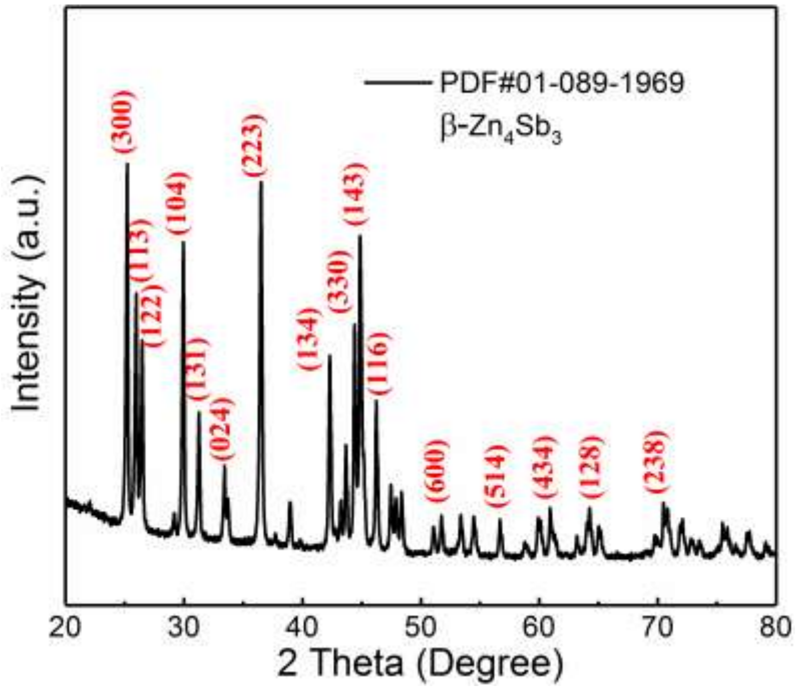

Figure S1. The powder XRD of β-Zn_4Sb_3 sample.

Table S1. Standard reaction quantities at 298.15 K for the compounds per mole atoms.[1]

Compound	T (K)	$\Delta_f H^\circ$ (J·mol^{-1})	$\Delta_f S^\circ$ (J·mol^{-1}·K^{-1})	$\Delta_f G^\circ$ (J·mol^{-1})
**ZnTe**	298.15	-117000.0	-7.492	-114766.0
**SnTe**	298.15	-60668.0	-1.659	-60173.4
**ZnSb**	298.15	-11543	-5.00	-10052

Gibbs free energy calculation:

SnTe + ZnSb → Sb@ZnTe + Sn

According to the data shown in Table S1, the Gibbs free energy of the above reaction was calculated as follows:

$$\Delta G = \Delta_f G^\circ(\text{ZnTe}) - \Delta_f G^\circ(\text{SnTe}) - \Delta_f G^\circ(\text{ZnSb})$$

$$= -114766.0 - (-60173.4-10052) = -44540.6 \text{ J·mol}^{-1} < 0$$

which implies that the reaction is favorable in thermodynamics.

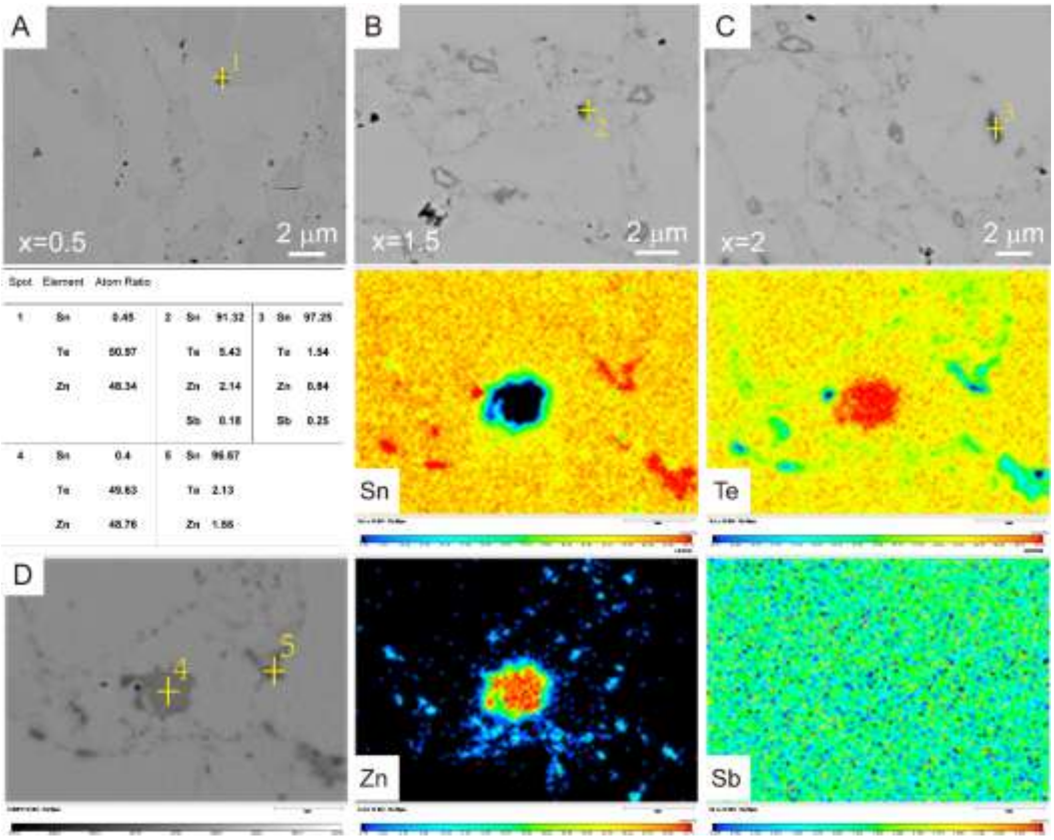

Figure S2. Backscattering scanning electron (BSE) microscopy images of SnTe-x at% (x=0.5, 1.5 and 2) β-Zn_4Sb_3 samples (A-C); EPMA element mapping results of the SnTe-1.5% β-Zn_4Sb_3 sample (D). The table show the point composition analysis results from the marked spot 1 to spot 5, which implies that the chemical composition of spot 1 and spot 4 are ZnTe nanoprecipitates while spot 2, 3 and 5 are Sn phase.

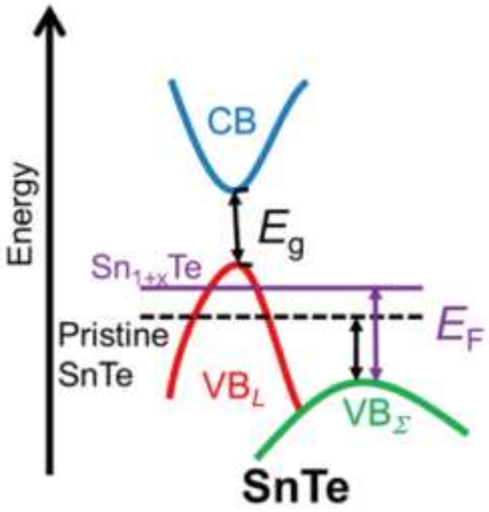

Figure S3. Schematic energy diagram of the electronic structure of SnTe near the Fermi level (E_F). The relative positions of the conduction band (CB), light hole valence band (VB_L), and heavy hole valence (VB_Σ) band are shown for pristine SnTe and Sn-compensated $Sn_{1+x}Te$. (Quoted in reference 3)[2]

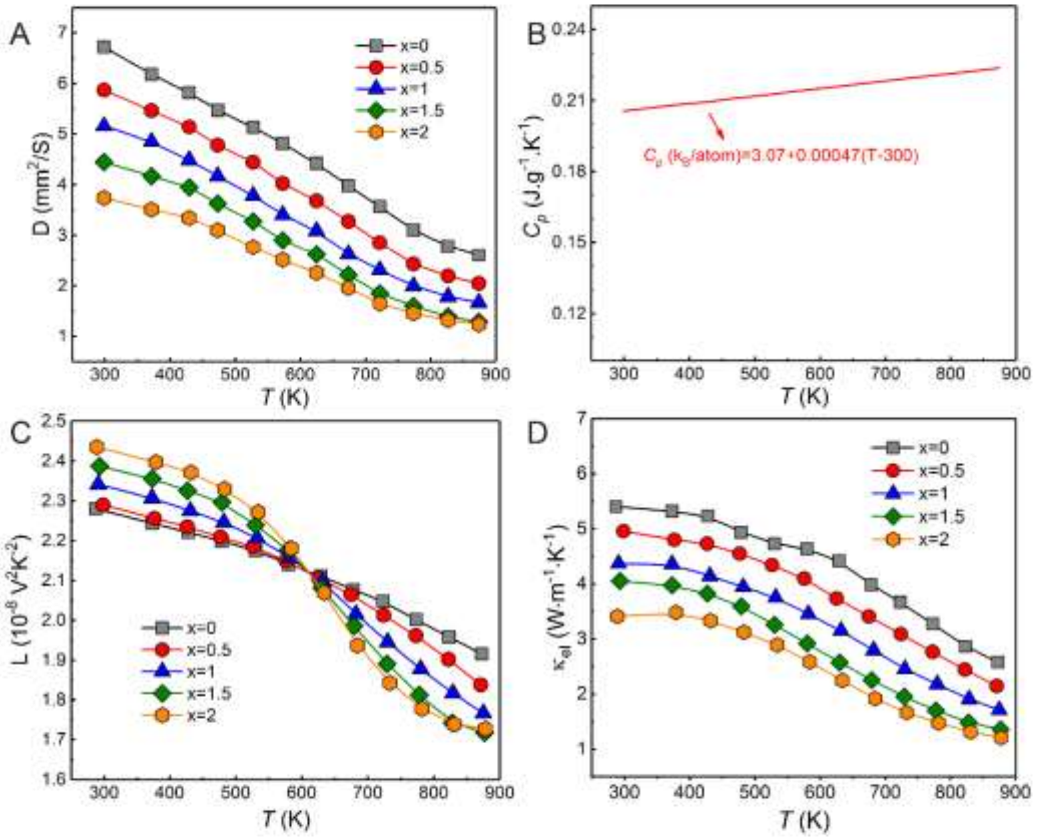

Figure S4. Temperature dependent thermal diffusivity (A); heat capacity (B); Lorenz number (C) and electronic thermal conductivity (D) of SnTe-x% β-Zn_4Sb_3 (x=0, 0.5, 1, 1.5 and 2) samples.

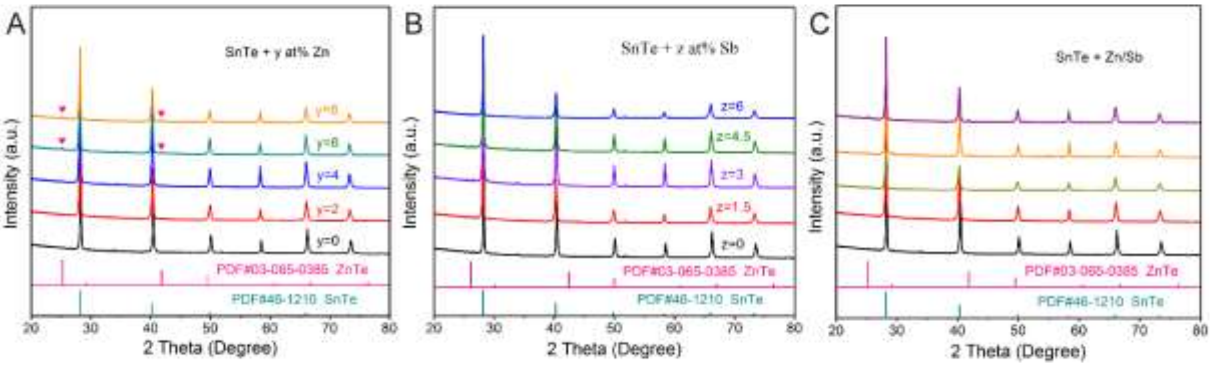

Figure S5. XRD patterns of SnTe-y% Zn (y=0, 2, 4, 6 and 8) samples (A); SnTe-z% Sb (z=0, 1.5, 3, 4.5 and 6) samples (B) and SnTe-Zn/Sb (0, 2/1.5, 4/3, 6/4.5 and 8/6) co-doped samples (C).

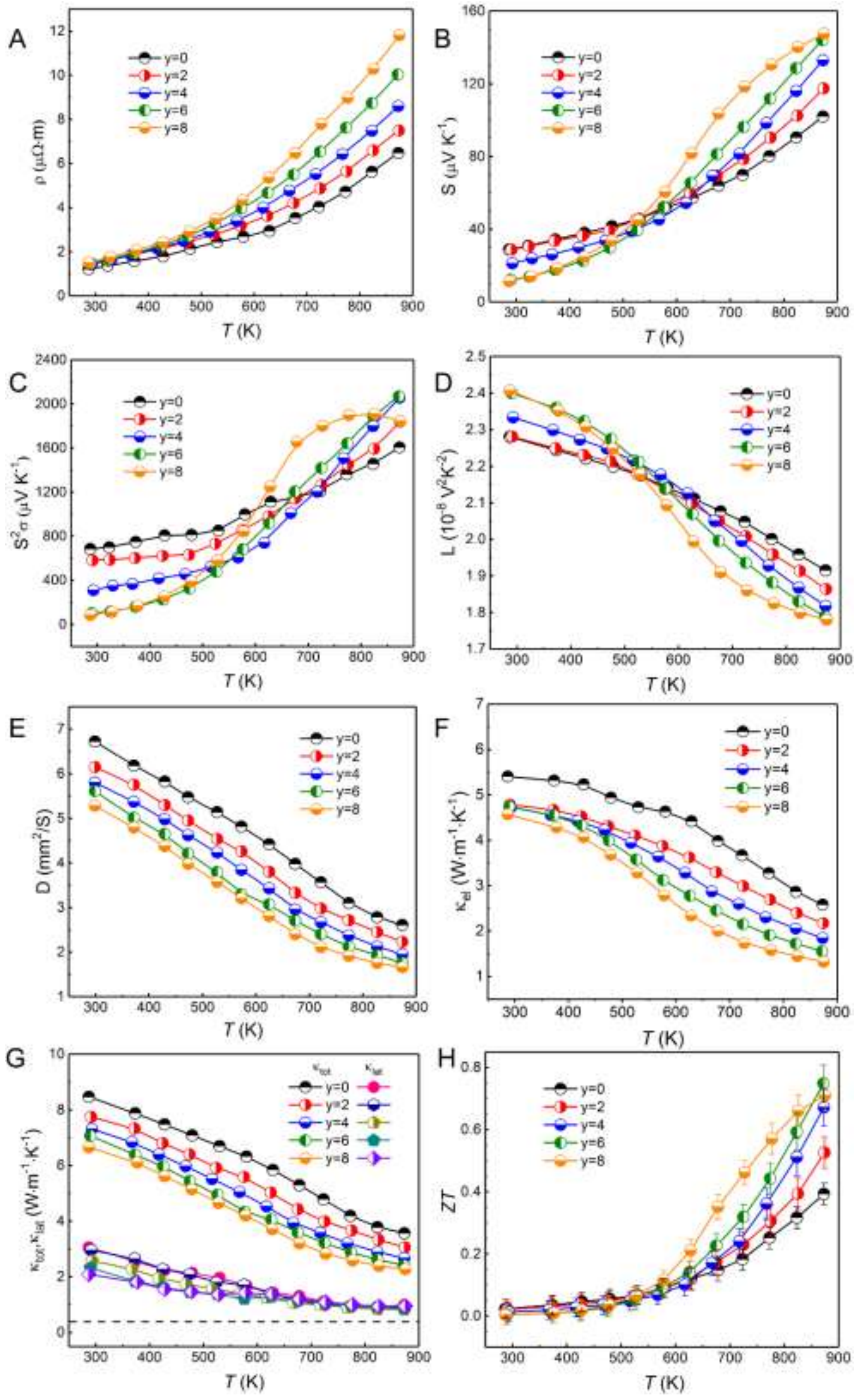

Figure S6. Thermoelectric properties for SnTe-y% Zn (y=0, 2, 4 ,6 and 8) samples. Temperature dependent electrical conductivity (A); Seebeck coefficient (B); power factor (C); Lorenz number (D); thermal diffusivity (E); electronic thermal conductivity (F); total and lattice thermal conductivities (G) and ZT values (H).

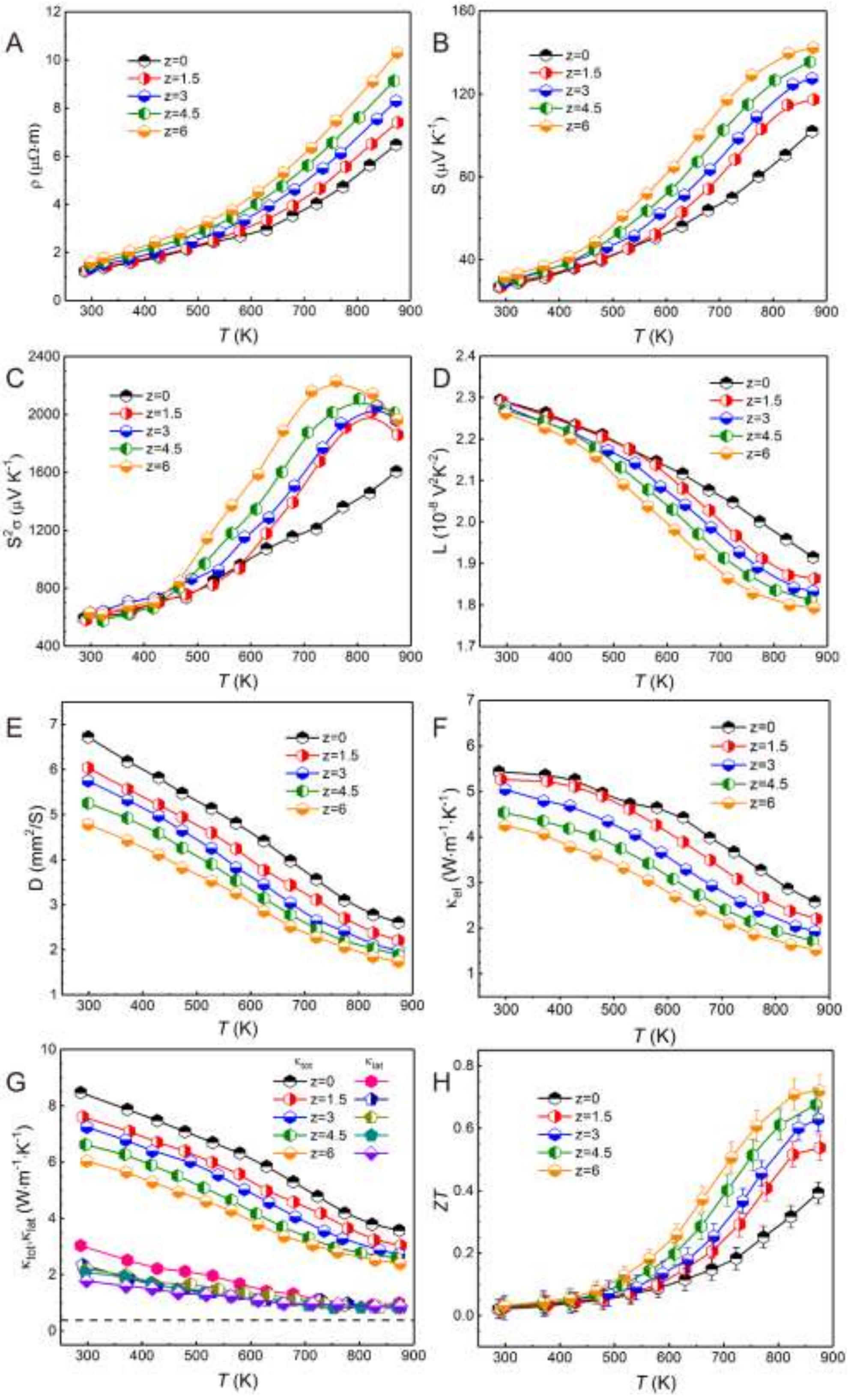

Figure S7. Thermoelectric properties for SnTe-z% Zn (z=0, 1.5, 3, 4.5 and 6) samples. Temperature dependent electrical conductivity (A); Seebeck coefficient (B); power factor (C); Lorenz number (D); thermal diffusivity (E); electronic thermal conductivity (F); total and lattice thermal conductivities (G) and ZT values (H).

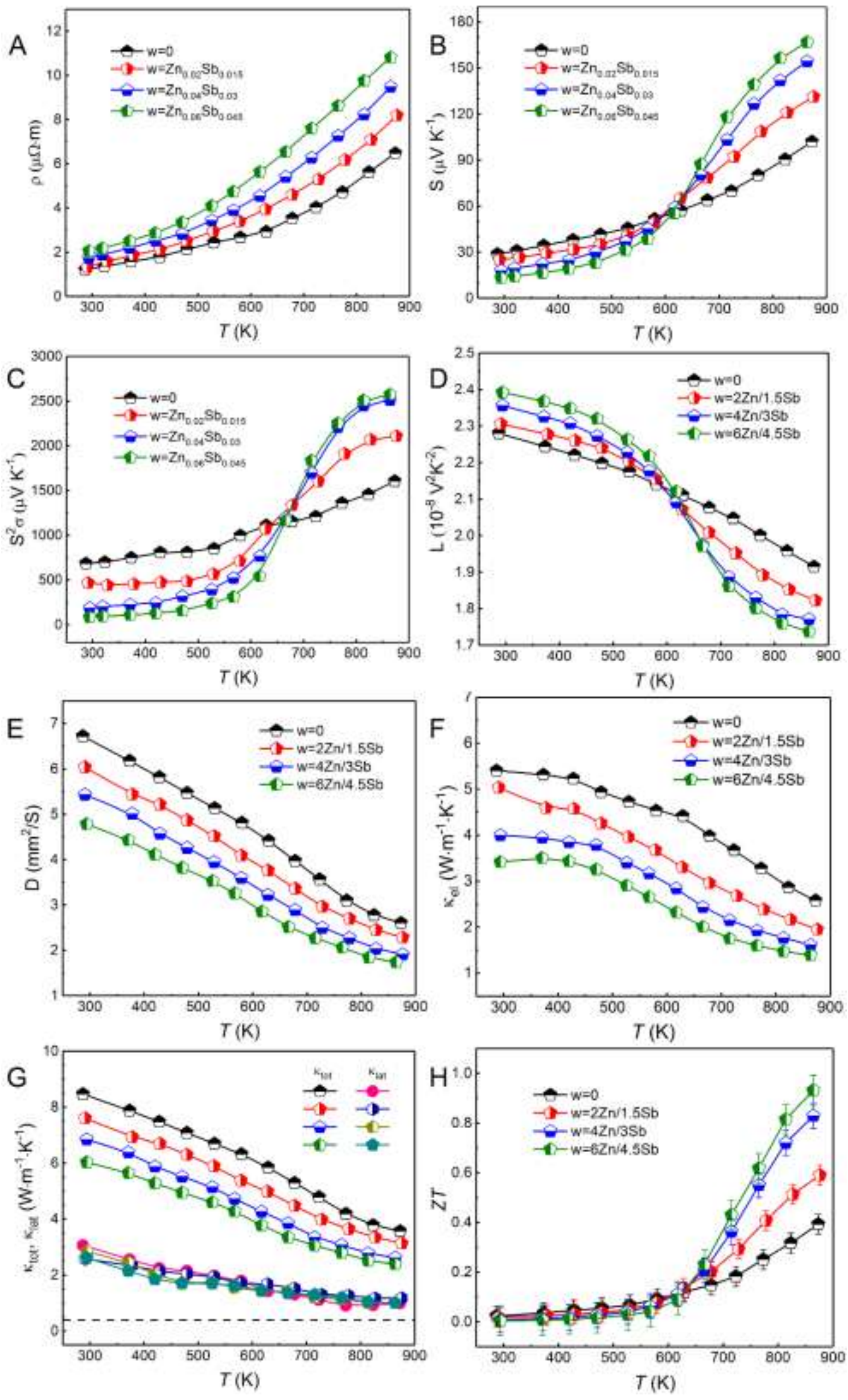

Figure S8. Thermoelectric properties for SnTe-Zn/Sb (2/1.5, 4/3 and 6/4.5) co-doped samples. Temperature dependent electrical conductivity (A); Seebeck coefficient (B); power factor (C); Lorenz number (D); thermal diffusivity (E); electronic thermal conductivity (F); total and lattice thermal conductivities (G) and ZT values (H).

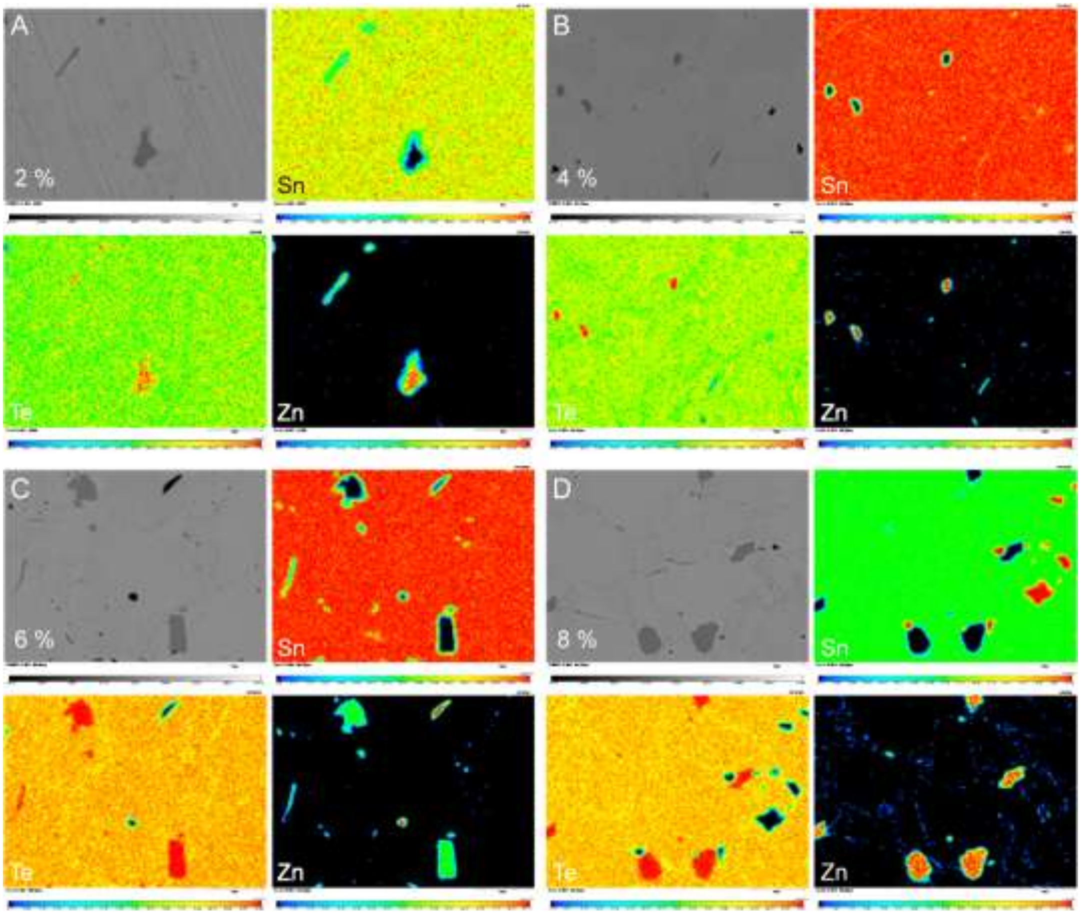

Figure S9. EPMA element analysis of (A) SnTe-2% Zn sample; (B) SnTe-4% Zn sample; (C) SnTe-6% Zn sample and (D) SnTe-8% Zn sample.

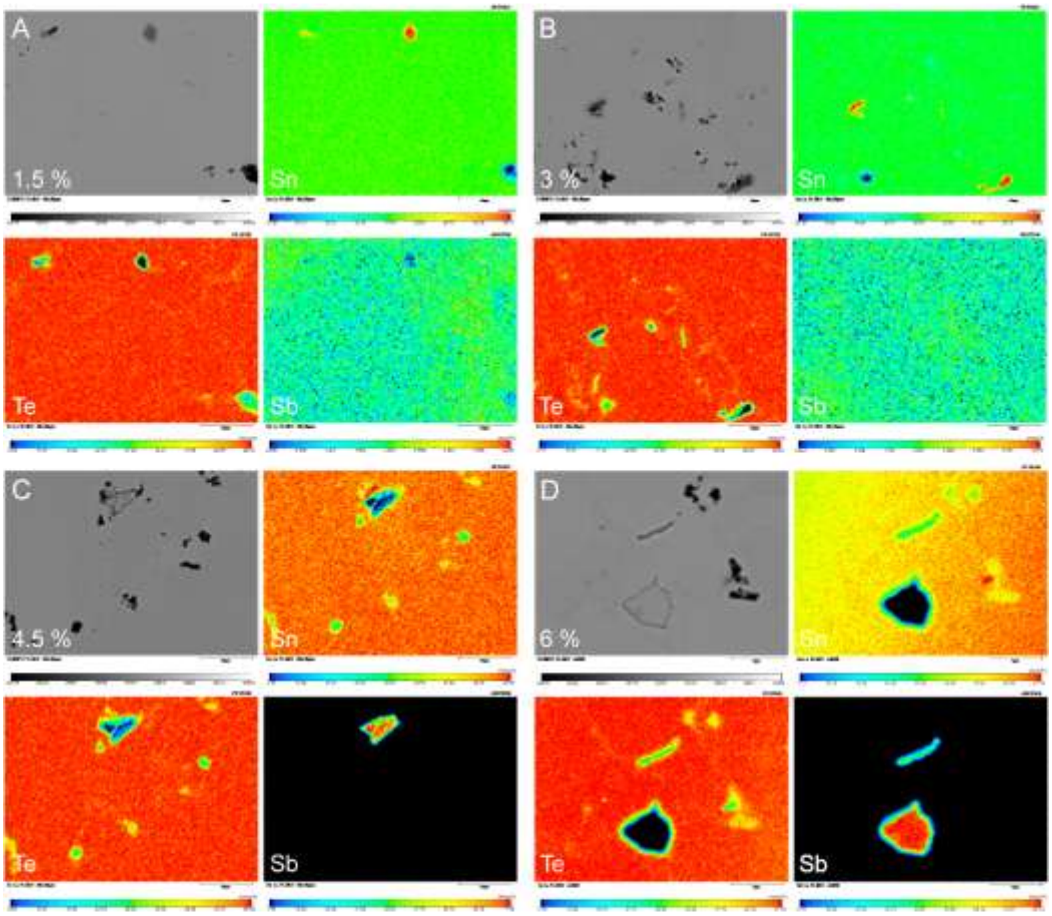

Figure S10. EPMA element analysis of (A) SnTe-1.5% Sb sample; (B) SnTe-3% Sb sample; (C) SnTe-4.5% Sb sample and (D) SnTe-6% Sb sample.

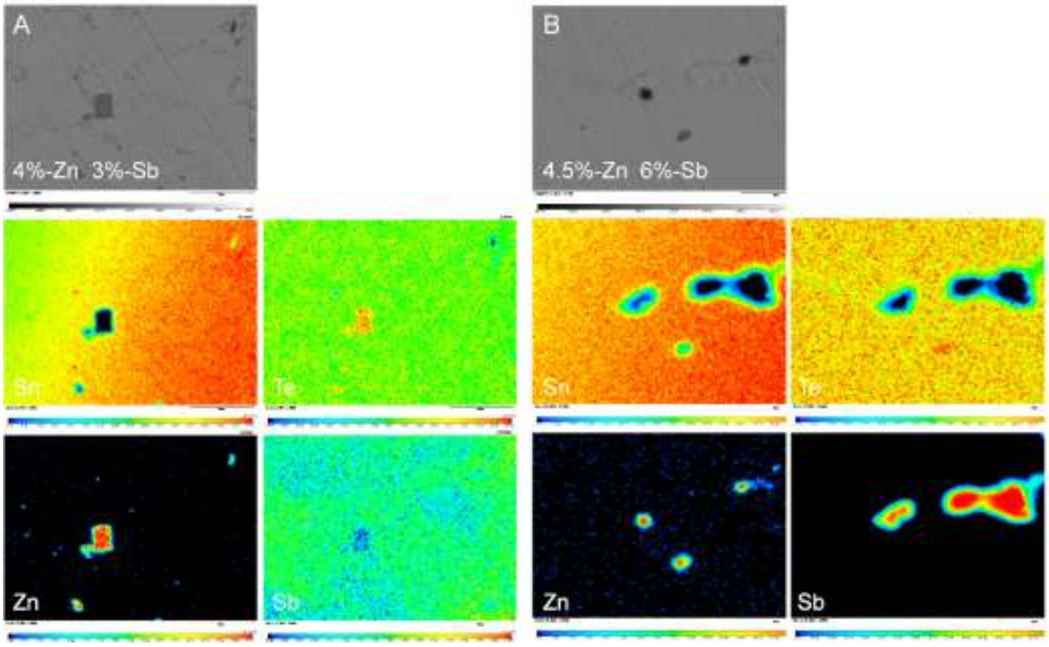

Figure S11. EPMA element analysis of (A) SnTe-4% Zn-3% Sb sample and (B) SnTe-6% Zn-4.5% Sb sample.

Note: Debye -Callaway model

In the Debye-Callaway model the lattice thermal conductivity is[3]:

$$\kappa_L = \frac{k_B}{2\pi^2 v}\left(\frac{k_B}{\hbar}\right)^3 T^3 \int_0^{\theta_D/T} \tau(x)\frac{x^4 e^x}{(e^x-1)^2}dx \qquad (4)$$

Where k_B, v, ħ, T, θ_D, and τ are the Boltzmann constant, the phonon velocity, the reduced Planck constant, the absolute temperature, the Debye temperature, and the phonon relaxation time, respectively. *x* is defined as $x = \hbar\omega/k_B T$ (ω is the phonon frequency).

The evaluation of phonon relaxation time follows the Matthiessen's rule,

$$\tau^{-1} = \tau_U^{-1} + \tau_{GB}^{-1} + \tau_{PD}^{-1} + \tau_D^{-1}\tau_{IT}^{-1} \qquad (5)$$

where the subscripts U, EP, PD, GB, and NP represent the Umklapp process, electron phonon scattering, point defect scattering, grain boundary scattering, and nanopartivles scattering, respectively.

The relevant phonon relaxation times are given by

Umklapp phonon scattering

$$\tau_U^{-1} = \frac{\hbar\gamma^2 x^2 T}{Mv^2\theta_D}\left(\frac{k_B T}{\hbar}\right)^2 exp\left(-\frac{\theta_D}{3T}\right) \qquad (6)$$

Electron phonon scattering

$$\tau_E^{-1} = \frac{{E_{def}}^2 m^{*2}\frac{k_B T}{\hbar}xT}{2\pi\left(\frac{h}{2\pi}\right)^3 \rho V_S \theta_D} * c \qquad (7)$$

Point defect phonon scattering

$$\tau_{PD}^{-1} = \left(\frac{k_B T}{\hbar}\right)^4 \frac{V_{atom}\Gamma}{4\pi v^3}x^4 \qquad (8)$$

Grain boundary phonon scattering

$$\tau_{GB}^{-1} = \frac{v}{L} \qquad (9)$$

Nanoparticles scattering

$$\tau_{NP}^{-1} = v({\sigma_s}^{-1} + {\sigma_l}^{-1})V_p \qquad (10)$$

Where $\sigma_S = 2\pi R^2, \sigma_l = \frac{4}{9}\pi R^2\left(\frac{\Delta D}{D}\right)^2\left(\frac{k_B T}{\hbar}x * \frac{R}{v}\right)^4$, V_p, R, D, M, γ, V_{atom}, and L are the number density of precipitates, average size of precipitates, theoretical density of precipitates, the average atomic mass, Grüneisen parameter, average atomic volume, and average grain size, respectively[4]. All these related parameters are listed in Table S2.

Table S2 Parameters for phonon modelling studies

**Parameters**	**Values**
Gruneisen parameter γ	2.3
Sound velocity *v*	1709m/s
Debye temperature θ_D	161 K
Average atomic mass M	$2.02*10^{-25}$ Kg
Grain size L	5 μm (SEM)
Average atomic volume V_{atom}	$3.125*10^{-29}$ m^{-3}
Lattice parameter	6.301 Å
Deformation potential E_{def}	35 eV (Fitted)
Average size of precipitates R	500 nm (SEM)
Number density of precipitates V_p	$9.6*10^{19}$ (TEM)
Density difference between matrix and precipitates ΔD	0.28 g cm^{-3}

References

[1] a) DOI: 10.1007/10688868_19, Springer-Verlag Berlin Heidelberg; b) P. Franke, D. Neuschütz, DOI: 10.1007/10757285_63 (Eds: P. Franke, D. Neuschütz), Springer-Verlag Berlin Heidelberg.

[2] G. Tan, L.-D. Zhao, F. Shi, J. W. Doak, S.-H. Lo, H. Sun, C. Wolverton, V. P. Dravid, C. Uher, M. G. Kanatzidis, *J. Am. Chem. Soc.* **2014**, 136, 7006.

[3] J. Callaway, *Physical Review* **1959**, 113, 436.

[4] R. D. Schmidt, E. D. Case, R. M. Trejo, E. Lara-Curzio, R. J. Korkosz, M. G. Kanatzidis, *J. Mater. Sci.* **2013**, 48, 8244.
